# Clinical review of high-flow nasal oxygen therapy in human and veterinary patients

**DOI:** 10.3389/fvets.2023.1070881

**Published:** 2023-03-06

**Authors:** Joanna Whitney, Iain Keir

**Affiliations:** Department of Emergency and Critical Care, Small Animal Specialist Hospital, Sydney, NSW, Australia

**Keywords:** high-flow nasal oxygen therapy (HFNOT), acute respiratory failure (ARF), hypoxemia, carbon monoxide toxicity, brachycephalic obstructive airway syndrome (BOAS), positive end-expiratory pressure (PEEP)

## Abstract

Oxygen therapy is the first-line treatment for hypoxemic acute respiratory failure. In veterinary medicine this has traditionally been provided *via* mask, low-flow nasal oxygen cannulas, oxygen cages and invasive positive pressure ventilation. Traditional non-invasive modalities are limited by the maximum flow rate and fraction of inspired oxygen (FiO_2_) that can be delivered, variability in oxygen delivery and patient compliance. The invasive techniques are able to provide higher FiO_2_ in a more predictable manner but are limited by sedation/anesthesia requirements, potential complications and cost. High-flow nasal oxygen therapy (HFNOT) represents an alternative to conventional oxygen therapy. This modality delivers heated and humidified medical gas at adjustable flow rates, up to 60 L/min, and FiO_2_, up to 100%, *via* nasal cannulas. It has been proposed that HFNOT improves pulmonary mechanics and reduces respiratory fatigue *via* reduction of anatomical dead space, provision of low-level positive end-expiratory pressure (PEEP), provision of constant FiO_2_ at rates corresponding to patient requirements and through improved patient tolerance. Investigations into the use of HFNOT in veterinary patients have increased in frequency since its clinical use was first reported in dogs with acute respiratory failure in 2016. Current indications in dogs include acute respiratory failure associated with pulmonary parenchymal disease, upper airway obstruction and carbon monoxide intoxication. The use of HFNOT has also been advocated in certain conditions in cats and foals. HFNOT is also being used with increasing frequency in the treatment of a widening range of conditions in humans. Although there remains conflict regarding its use and efficacy in some patient groups, overall these reports indicate that HFNOT decreases breathing frequency and work of breathing and reduces the need for escalation of respiratory support. In addition, they provide insight into potential future veterinary applications. Complications of HFNOT have been rarely reported in humans and animals. These are usually self-limiting and typically result in lower morbidity and mortality than those associated with invasive ventilation techniques.

## 1. Introduction

**Figure 1:**
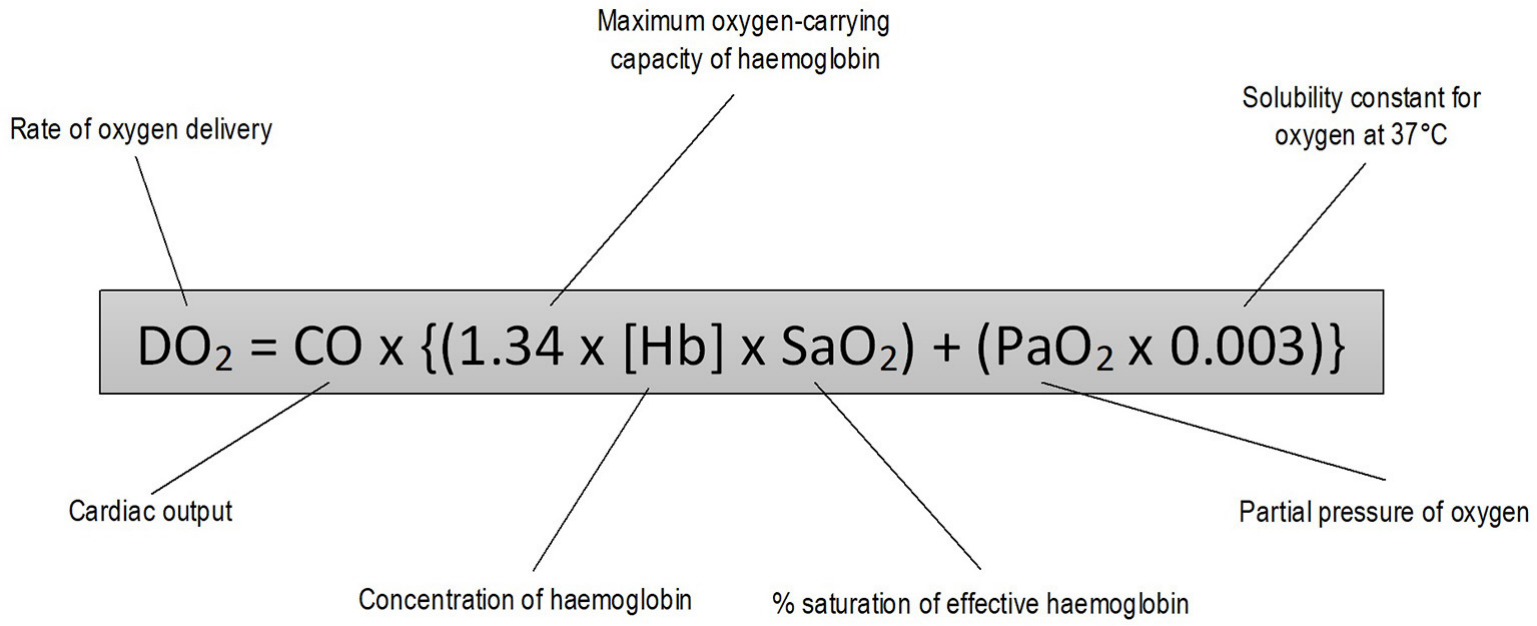
Oxygen delivery equation.

Oxygen delivery to tissues depends on adequate ventilation, gas exchange and circulatory distribution (Figure 1) (1). Conditions resulting in tissue hypoxia can be classified into three groups: (i) those causing arterial hypoxemia [decreased fraction of inspired oxygen (FiO_2_), alveolar hypoventilation, gas diffusion impairment, ventilation-perfusion (V/Q) mismatch, cardiopulmonary shunt]; (ii) those causing failure of the oxygen-hemoglobin transport system without arterial hypoxemia (anemia, dyshemoglobinemia, and inadequate blood flow to tissues) and (iii) those impairing the cells' ability to utilize oxygen (cyanide toxicity) (2).

The cornerstone of treatment of hypoxemic patients is the provision of increased FiO_2_ to improve oxygen delivery by ensuring oxygen saturation of hemoglobin and increasing the concentration of dissolved oxygen in plasma (1). Oxygen delivery systems are categorized into low-flow and high-flow systems. Low-flow systems deliver oxygen at a flow rate that is lower than the patient's ventilatory requirements resulting in dilution of the concentration of inspired oxygen relative to the inspiratory flow rate. Modalities in this category are generally considered conventional oxygen therapy (COT). High-flow systems can provide gas at a rate to match minute ventilation and, therefore, a stable and predictable FiO_2_ (3). A variety of oxygen delivery systems have been described in human and veterinary medicine (Table 1).

**Table 1:** Modes of oxygen supplementation in veterinary patients.

	**FiO_2_ (%)**	**Flow rate**	**Advantages**	**Limitations**	**Indications**
**Low flow**					
Flow by	25–45	6–8 L/min	• Utilizes readily available equipment	• Not appropriate for prolonged therapy •Wasteful	• Triage and procedures • Initial stabilization
Oxygen cage	21–60		• Well tolerated • Allows eating and drinking	• Reduced access to patients • FiO_2_ rapidly decreases when doors opened • Larger patients	• Patients that will not tolerate nasal oxygen or in which nasal oxygen is contraindicated
Face mask	35–55	1–6 L/min	• Utilizes readily available equipment • Rebreathing at low rates	• Not appropriate for prolonged therapy • FiO_2_ depends on fit of mask	• Triage and procedures • Initial stabilization • Risk of rebreathing
Nasal prongs		50–150 ml/kg/min	• Easy to place • Well tolerated	• Poor patient tolerance at high flow rates • Not suitable for some facial conformations	• Ongoing oxygen support in hospital
Nasal catheter	30–60	50–150 ml/kg/min	• Well tolerated	• Poor patient tolerance at high flow rates • Harder to place	• Ongoing oxygen support in hospital
**High flow**					
CPAP	21–100		• Reliable FiO_2_ • Delivers PEEP • Humidifies inhaled gases	• Often requires heavy sedation • Specific equipment	• Hypoxaemia despite oxygen support • Upper airway obstruction
HFNOT	21–100	10–60 L/min	• Reliable FiO_2_ • Delivers PEEP • Humidifies inhaled gases	• Specific equipment	• Hypoxaemia despite conventional oxygen therapy • Increased work of breathing
Mechanical ventilation	21–100		• Reliable FiO_2_ • Delivers PEEP • Humidifies inhaled gases	• Specific equipment • High complication rate • High cost	• Hypoventilation • Hypoxaemia despite oxygen support • Increased work of breathing (fatigue)

Oxygen cages are frequently used in small animal patients to provide oxygen at a set FiO_2_, humidity and temperature while efficiently removing carbon dioxide (2). However, the oxygen levels within the cage rapidly deplete when the doors and opened, complicating patient monitoring and treatments.

Flow-by oxygen is a simple technique often applied in emergency veterinary patients. Positioning an oxygen line within a few centimeters of a patient's nostrils creates a small area of available air with increased oxygen concentration (2). A oxygen flow rate of 2–3 L/min may provide a FiO_2_ of 25–40% (4).

In humans low flow masks can provide up to 60% FiO_2_ at moderate flow rate (6–10 L/min). However, these need to be tight-fitting to prevent entrainment and significant rebreathing may occur at flow rates <5 L/min (1). In veterinary patients, a face mask positioned over the muzzle can be used during initial stabilization or in immobile patients, however ongoing use in conscious patients is limited by a lack of compliance. The FiO_2_ generated and degree of rebreathing that occurs depends on the tightness of fit of the mask as well as the oxygen flow rate (4).

Nasal prongs provide low-flow oxygen with improved comfort compared to mask oxygen. The FiO_2_ depends on the oxygen flow rate and varies according to minute volume. In resting human patients, 2 L/min oxygen results in 25–30% nasopharyngeal oxygen (1). Although flow rates >6 L/min can be achieved with some systems, these should be avoided as they are associated with dry of the nasal mucosa (3). Nasal prongs prevent rebreathing and allow patients to eat. Human nasal prongs can be used in larger dogs of suitable conformation, however no investigations of the efficacy of this technique have been undertaken.

Nasal oxygen catheters are typically used in place of nasal prongs to provide ongoing low flow oxygen support in veterinary patients. Higher FiO_2_ can be achieved compared to flow-by and mask interfaces but is dependent on oxygen flow rate, the degree of open-mouth breathing of the patient and if unilateral or bilateral cannulas are placed (2). Dunphy et al. demonstrated that mean tracheal FiO_2_ of 77% can be achieved with flow rates of 200 ml/kg/min delivered bilaterally in healthy dogs (5). However, flow rates > 100 ml/kg/min resulted in discomfort and distress in this group of patients; while 100 ml/kg/min administered bilaterally were well tolerated and resulted in a mean FiO_2_ of 56% (5).

High-flow mask oxygen utilizes Venturi valves to create a Bernoulli effect and provide higher flow rates (30–50 L/min) generating 24–60% FiO_2_. These systems are able to provide the total ventilatory requirement of the patient regardless of the pattern of ventilation and eliminate rebreathing due to the high flow rate (1). However, humidification is limited to a standard bubble humidifier resulting in inadequate humidification of inhaled gases and subsequent airway desiccation and discomfort (6, 7).

Non-invasive ventilation refers to the delivery of mechanical ventilation *via* techniques that do not require endotracheal intubation (8). These improve gas exchange and reduce inspiratory effort through the generation of positive pressure within the airways, reducing upper airway obstruction and recruitment of alveoli (9). There are two main modalities of non-invasive ventilation—continuous positive airway pressure (CPAP) and non-invasive pressure support ventilation (10). CPAP may be performed using a tight-fitting mask or helmet with an expiratory (positive end-expiratory pressure, PEEP) valve connected to an oxygen source and gas blender or *via* a mechanical ventilator. Non-invasive pressure support ventilation requires a ventilator triggered by the patient's inspiratory effort to deliver a decelerated gas flow in order to generate and maintain two different pre-set pressures during inspiration and expiration (10). Nasal or oronasal (full face) masks which form an air seal are required to achieve non-invasive pressure support ventilation (8, 11). Non-invasive ventilation *via* nasal mask in cats requires similar levels of sedation as and confers no significant cardiovascular benefits compared to mechanical ventilation (11).

Significant increases in PaO_2_ have been demonstrated after CPAP administered by helmet in dogs (pre-CPAP 80.6 mmHg vs. CPAP 105.6 mmHg) and anesthetized cats (pre-CPAP 77.5 mmHg vs. CPAP 103.2 mmHg) (12, 13). Raidal et al. compared the effect on respiration and ventilation in sedated foals treated with CPAP and mask oxygen (14). They found that the effects of CPAP on arterial blood gas parameters were comparable to mask oxygen with modest increases in PaCO_2_ in almost all animals for both modalities. The clinical use of CPAP in veterinary patients is effective in providing a known level of PEEP and improving oxygenation. However, its use is currently limited by the need for sedation or anesthesia for the interface to be tolerated in some patients and the high oxygen flow requirement to maintain PEEP (13, 15).

Invasive mechanical ventilation is indicated in the management of severe hypoventilation, severe hypoxemia despite oxygen supplementation, when there is excessive work of breathing and when long-term endotracheal intubation is required (16). ICU ventilators that can provide humidified gas up to 100% FiO_2_ are recommended for the management of such conditions. In addition to oxygen support and conditioning of inspired gases, mechanical ventilation reduces the work of breathing and can improve oxygenation *via* increasing airway and alveolar pressure and recruiting collapsed alveoli (16).

Heavy sedation or a light plain of anesthesia is generally required to facilitate mechanical ventilation (16). A number of potential complications have also been reported in veterinary patients associated with mechanical ventilation including corneal and oral mucosal ulceration, hypothermia, positive fluid balance, ventilator-induced lung injury, ventilator-associated pneumonia and cardiovascular compromise (17–19). The prognosis for veterinary patients undergoing mechanical ventilation for conditions other than anesthesia-associated hypoventilation is variable and depends on the underlying condition (16). Overall, patients ventilated for primary hypoventilation have a better prognosis that those being treated for primary pulmonary pathology, in particular acute respiratory distress syndrome (ARDS) (17, 20). Intensive patient care and specialized equipment is required to provide effective mechanical ventilation and minimize complications, and is associated with high cost to clients (21).

**Figure 2:**
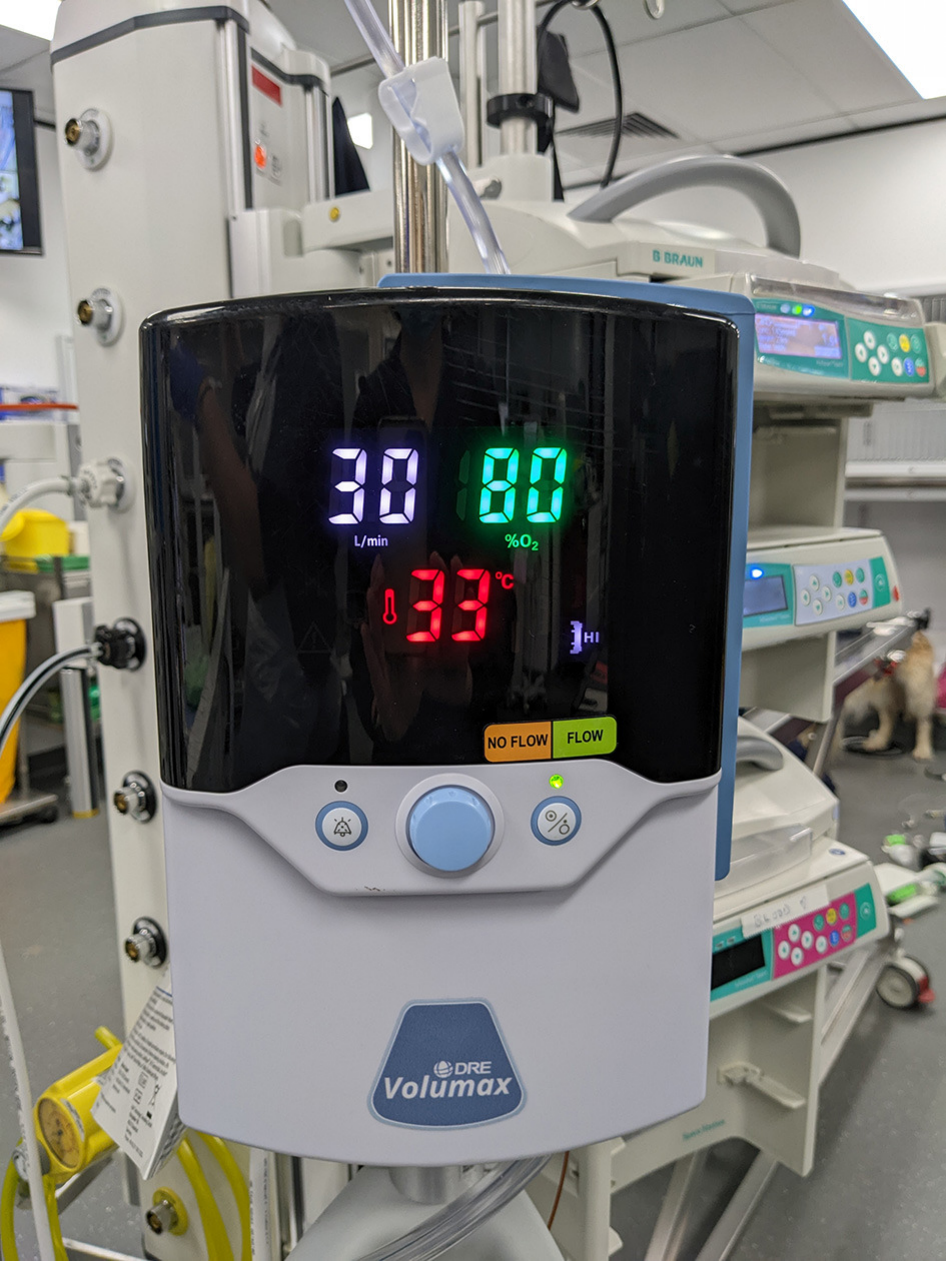
HFNOT device on which flow rate, FiO_2_, and temperature can be set.

High-flow nasal oxygen therapy (HFNOT) is a non-invasive respiratory support able to provide some of the features previously limited to mechanical ventilatory techniques. It involves the delivery of a humidified gas mixture [37°C contains 44 mg H_2_O (100% relative humidity)] at up to 60 L/min, with a FiO_2_ ranging from 21 to 100% (22). HFNOT devices allow modification of three variables—the percentage of oxygen delivered, the flow rate of gas and gas temperature (Figure 2). Components of HFNOT devices include: a high pressure sources of oxygen and air, an air-oxygen blender or a high-flow “Venturi” system, a humidifying and heating system for conditioning the gas to optimal temperature and humidity, a sterile water reservoir, a non-condensing circuitry, and an interface (6).

There are several devices available which provide high flow, humidified oxygen *via* a nasal cannula. The Precision Flow^TM^ (Vapotherm) and Optiflow^TM^/AirFlo 2^TM^ (Fischer and Paykel) are the most commonly used in human medicine and veterinary reports (6, 23–30). An increasing number of HFNOT systems have become available in the last 3–5 years. A recent benchtop study found a marginal but statistically significant difference in key performance parameters, however, the clinical effect of these differences has not yet been evaluated *in vivo* (31).

Relevant studies published until June 2022 were retrieved from the online databases Web of Science and PubMed using the keywords “high-flow oxygen” and “high-flow” AND “oxygen.” Reference lists of search articles were reviewed for additional relevant articles. All veterinary articles and human articles, with the highest level of evidence for the topic, deemed relevant and/or applicable to veterinary medicine were reviewed.

## 2. Mechanisms of action and proposed physiological benefits of high-flow nasal oxygen therapy

A number of physiological mechanisms have been proposed to explain the beneficial effects of HFNOT in human medicine, however some aspects have not been fully investigated. Furthermore, the contribution of each mechanism in different clinical conditions and individual patients likely varies and these have yet to be fully elucidated.

Clinically relevant features of HFNOT include provision of fixed concentrations of inspired gases, delivery of heated and humidified gases, generation of flow-dependent positive airway pressure and flushing of anatomical dead space. These features result in increased patient comfort and compliance, provision of higher FiO_2_ compared to conventional oxygen therapy, maintenance of mucosal integrity and function, alveolar recruitment and decreased work of breathing (9, 32, 33).

### 2.1. Respiratory mechanics and oxygenation

#### 2.1.1. FiO_2_ and dead space washout

The flow rates provided during HFNOT, which are higher than those achieved using conventional oxygen therapy, are able to match the peak inspiratory flow of the patient, and thus reliably provide the set FiO_2_ (34, 35). As such, FiO_2_ approaching 100% are able to be achieved without the need for endotracheal intubation. Higher oxygen flow rates delivered during HFNOT compared to conventional oxygen therapy are also proposed to “washout” carbon dioxide from the anatomical dead space within the nasopharynx resulting in reduced rebreathing. This results in an improved FiO_2_, more efficient provision of minute ventilation and decreased work of breathing (32, 33, 36–38).

Carbon dioxide washout has also been proposed to contribute to the mechanism by which HFNOT is effective in sleep breathing disorders. Reduced levels of inspired carbon dioxide may improve breathing patterns in these patients (33).

#### 2.1.2. Positive airway pressure

The provision of PEEP during any form of respiratory support aims to prevent alveolar collapse and recruit atelectatic lung, ultimately improving alveolar ventilation (16). Additionally, providing higher flow rates to match intrinsic PEEP *via* CPAP reduces the work of breathing in patients with obstruction airway disease (39–41).

A small study of human cardiac surgery patients demonstrated a positive linear relationship between flow rate and mean airway pressure during HFNOT, with PEEP ranging from 3.0 to 4.8 cmH_2_O at flow rates of 30–50 L/min (42). The authors proposed that this PEEP resulted, in part, from the resistance to expiration associated with the continuous incoming gas flow (43). Studies in which pharyngeal and esophageal pressures have been measured report generation of a 2–4 cm H_2_O in children (44, 45).

Individual variations in the generation of airway pressure during HFNOT have been identified in a number of studies and attributed to anatomical and physiological differences between patients (43, 46, 47). Of particular consideration are the diameter of the nasal prongs relative to the patient's nares and whether or not the mouth is closed (43). Multiple prospective studies of adult humans treated with HFNOT have demonstrated that significant airway pressure generation only occurs when the patient's mouth is closed (34, 43, 46).

#### 2.1.3. Airway resistance

An important function of the nasopharynx is to facilitate warming and humidification of inspired gases by contact with its large surface area. However, this function also results in significant resistance to inspiratory flow (48). By matching or exceeding rates of inspiratory flow, HFNOT likely attenuates this effect and further reduces the work of breathing (33).

A pharyngeal distending pressure of up to 4 cm H_2_O can be achieved with HFNOT flow rates of 2 L/kg/min (44). This positive upper airway pressure may reduce airway resistance by stenting the soft palate and pharynx (44). It has also been proposed that HFNOT stimulates airway stiffening and stenting by activation of the alae nasae muscle (49).

HFNOT may also alleviate increased resistance in the lower airways that can result from conventional oxygen therapy. Provision of cool, dry air during respiratory support has been shown to decreased pulmonary compliance and conductance in infants (50). This has been demonstrated to be associated with a protective bronchoconstrictive response, secondary to stimulation of muscarinic receptors in the nasal mucosa, in healthy and asthmatic children (51–53). Clinically, this has been supported by Saslow et al. who demonstrated beneficial effects of the provision of warm, humidified air *via* nasal catheter in infants (54). In this study, pulmonary compliance was higher in patients receiving HFNOT compared to CPAP, despite the former providing lower PEEP.

In addition to the increased resistance created in the upper respiratory tract, the conditioning of inhaled gases by the nasal mucosa also consumes energy (33). This energy requirement is conceivably increased during supplementation of cool, dry gas during conventional oxygen therapy as well as during periods of respiratory distress and increased minute ventilation (33).

### 2.2. Mucociliary clearance

Mucociliary clearance is an import defense mechanism of the airways and depends on normal cilia function and mucus composition (55, 56). Slow, turbulent airflow in the nasopharynx allows inspired air to be warmed to ~34°C and humidified to 100%. This helps to create optimal conditions for the functioning of cilia and maintenance of mucus composition (57). The formation of respiratory secretions also depends on adequate moisture content of the respiratory epithelium.

Patient and treatment factors may affect conditioning of inspired air and mucociliary clearance. Increased respiratory rates and open-mouth breathing in respiratory failure can affect airway humidification and conditioning of inspirated gas (58). HFNOT has been shown to reduce respiratory rates and effort, and may be able provide clinical improvement through the reduction in open-mouth breathing (59).

The provision of cold, dry gas through conventional oxygen therapy further exacerbates the detrimental effects on inspired gas and airway function (60). An *in vitro* study of tracheal epithelium demonstrated that exposure to low-humidity inspired gas, even for relatively short periods, impaired the function of human epithelial cells. This may result in mucus dehydration, impaired mucociliary clearance and mucus retention (60). HFNOT delivers gas at 100% and close to body temperature, features that may be advantageous in maintaining or improving mucociliary clearance (61). This has been demonstrated in canine model in which provision of heated and humidified gas improved mucociliary function (62).

### 2.3. Patient comfort

HFNOT is better tolerated and more comfortable than conventional oxygen therapy (63, 64) and non-invasive ventilation (65–67). This has been attributed to a number of factors including conditioning of inspired gas, correction of hypoxemia, increased alveolar recruitment and the ability to eat and speak more readily (22).

#### 2.3.1. Conditioning of gas

As previously discussed, HFNOT maintains conditioning of inspired air and hydration of the airway mucosa. By not desiccating the nasal passages, HFNOT is proposed to provide more comfort and better tolerance of the higher flow of gas (33, 68–70). However, a recent pilot study by Spoletini et al. proposed that additional features of HFNOT may be associated with improved patient comfort (71).

#### 2.3.2. Device-patient interface

In humans, conventional oxygen therapy may be delivered by a face mask or nasal cannula while non-invasive ventilation utilizes oronasal masks, full-face masks or helmets. Non-invasive ventilation interfaces in particular are associated with the development of skin lesions and patient discomfort (72). Since the interface of nasal CPAP requires secure fixing without leaks, the reported incidence of pressure ulcers ranges from 15 to 100% (73). Furthermore, masks and helmets may interfere with eating and drinking (3). When compared to conventional nasal oxygen therapy patients, HFNOT patients experience decreased eye irritation and find it easier to eat (71).

## 3. High-flow nasal oxygen therapy in human medicine

### 3.1. Indications

#### 3.1.1. Acute hypoxemic respiratory failure

HFNOT is becoming the first-line therapy for acute hypoxemic respiratory failure in patients that fail to show an adequate response to conventional oxygen therapy and for whom immediate intubation is not indicated (74). In addition to its therapeutic benefits, a cross-over study demonstrated that this modality significantly reduced discomfort in critically ill patients with respiratory failure compared to conventional therapy (75).

The FLORALI clinical trial compared conventional oxygen therapy (≥10 L/min *via* mask), HFNOT (50 L/min) and non-invasive ventilation (≥8 h/day with bilevel settings) (76). This study included 310 non-hypercapnic patients with acute hypoxemic respiratory failure (P_a_O_2_/FiO_2_ < 300 mmHg), 84% of which had pneumonia. There was no significant difference in intubation rates between the three groups, however, mortality was lower in the HFNOT group in the ICU (11% with HFNOT vs. 19% with COT vs. 25% with NIV) and at 90-days (12% with HFNOT vs. 23% COT vs. 28% NIV). *Post-hoc* analysis demonstrated that HFNOT was associated with a decreased intubation rate in a more severely affected subgroup of patients (P_a_O_2_/FiO_2_ < 200 mmHg) (76).

More than 75% of patients in the FLORALI trial had thoracic radiographic changes that were consistent with a diagnosis of early ARDS (77). A contemporary observational study evaluated the effect of HFNOT in ARDS (78). Modes of oxygen support were considered in 607 patients admitted to a single medicosurgical ICU. 45/51 patients who received HFNOT as a first-line treatment had ARDS (P_a_O_2_/FiO_2_ 137 mmHg), with 26/45 successfully treated with HFNOT alone. Patients who failed HFNOT had higher Simplified Acute Physiology Score II (SAPS II) scores in multivariate analysis (78).

The HOT-ER trial aimed to determine if HFNOT compared to conventional oxygen therapy reduced the need for non-invasive ventilation or intermittent positive pressure ventilation in patients with acute respiratory distress presenting to a hospital emergency department (79). 322 hypoxemic (SpO_2_ ≤ 92%) and tachypneic (respiratory rate ≥ 22 breaths/min) adult patients were randomized to receive HNFOT or conventional oxygen therapy with need for mechanical outcome as the primary outcome. HFNOT did not reduce the need for ventilation in this population, results which conflicted with the FLORALI trial but may be reflective of the more heterogeneous population (76, 79). Of note, whilst adverse events were infrequent in the HOT-ER trial, 1/12 patients did not tolerate HFNOT (79).

Much of the recent human literature regarding HFNOT in acute hypoxemic respiratory failure has focused on its use in the treatment of COVID-19. HFNOT has been demonstrated to result in a significant reduction in intubation rate and subsequent mechanical ventilation without a clear survival benefit compared to conventional oxygen therapy (80–82). A randomized clinical trial of ICU patients with moderate to severe COVID-19 found that HFNOT was associated with higher rates of intubation compared to helmet non-invasive ventilation (83). Despite this, there was no difference in in-hospital mortality between groups in this study.

The Surviving Sepsis Campaign: Guidelines on the Management of Critically Ill Adults with Coronavirus Disease 2019 (COVID-19) suggest the use of HFNOT over conventional oxygen therapy in adults with acute hypoxemic respiratory failure despite conventional oxygen therapy. Furthermore, they suggest HFNOT over non-invasive positive pressure ventilation (84). At the time of their publication, prospective and randomized control trials investigating the use of HFNOT in the management of COVID-19 were scarce and these recommendations were based predominantly on the FLORALI trial (76).

#### 3.1.2. Acute heart failure

In addition to providing oxygen support, HFNOT has been proposed to have haemodynamic effects that may aid in the management of acute heart failure and pulmonary oedema. In particular, reducing pulmonary congestion *via* reductions in cardiac preload and afterload (85). Roca et al. performed echocardiographs in 10 patients with NYHA class III heart failure during HFNOT delivered at flow rates of 20 L/min and 40 L/min with 21% FiO_2_ (86). Significant reduction in inspiratory collapse of the inferior vena cava occurred relative to flow rate during HFNOT and normalized following discontinuation of therapy. The authors reasoned that as right atrial pressure is estimated by inferior vena cava diameter collapses, and the right atrium is a surrogate of right ventricular preload, HFNOT may be associated with a decrease in preload (86). Additionally, HFNOT may decrease afterload through the provision of PEEP and amelioration of sympathetic nervous system stimulation associated with hypoxia (87). Despite the proposed benefit of HFNOT in the management in acute left-sided cardiac failure, prospective studies of its clinical utility are limited.

A prospective randomized controlled study by Makdee et al. assessed the efficacy of HFNOT in the management of acute heart failure by comparing respiratory rates following HFNOT with conventional oxygen therapy in patients presenting to an emergency room with pulmonary oedema (88). Patients were included if they had SpO_2_ < 95% and respiratory rate >25 breaths/min; patients requiring ventilation, with the presence of myocardial infarcts or evidence of other organ failure were excluded. HFNOT was associated with lower respiratory rates at 60 mins after initiation of therapy but there was no difference in mortality, non-invasive ventilation or intubation between groups.

A more recent prospective study of HFNOT in the management of acute congestive heart failure compared HFNOT with conventional oxygen therapy in patients presenting to the emergency room with acute pulmonary oedema (89). The conventional oxygen therapy group received oxygen *via* a nasal cannula at flow rates of >2 L/min while HFNOT was initiated at 45 L/min and FiO2 100%. Patients in both groups were treated to maintain SpO_2_ > 93%. HFNOT resulted in greater improvement in respiratory rate, SpO_2_, lactate levels and arterial blood gas parameters compared with conventional therapy (89).

Osman et al. conducted a randomized controlled trial comparing HFNOT to non-invasive ventilation (helmet CPAP) in adult patients presenting the emergency room with acute cardiogenic pulmonary oedema (90). They found CPAP to be more effective in the very short term in improving dyspnoea, hemodynamics and respiratory parameters.

#### 3.1.3. Interstitial lung disease

The interstitial lung diseases are a heterogeneous group of pulmonary conditions that involve changes to the distal lung parenchyma (91). The different diseases can be subdivided into those with an identifiable etiology and those without, and have marked variation in regards to their clinical course, treatment and prognosis (92). Acute respiratory failure can complicate interstitial lung disease and is associated with a poor prognosis and high mortality rate (93). Mechanical ventilation does not improve oxygenation in affected patients and is associated with a high incidence of barotrauma and a poor prognosis (94, 95). HFNOT has been proposed as an alternate method of oxygen support and palliation in patients with interstitial lung disease. At this time, information is limited to case series and retrospective studies.

Horio et al. (96) reported three case of acute respiratory failure associated with interstitial lung disease. The patients were commenced on HFNOT (FiO_2_ 70–100%; flow rate 40 L/min) while additional medical management was initiated and took effect. HFNOT was well-tolerated and weaned in accordance with improving oxygenation parameters until discharge at 21–26 days (96). All of the subjects in this series demonstrated immediate improvement with the commencement of HFNOT after failing to respond to conventional oxygen therapy (96).

This initial support for HFNOT in the management of interstitial lung disease was supported by a retrospective study of 96 patients with exacerbation of interstitial pneumonia (97). Patients were grouped into pre-HFNOT and post-HFNOT cohorts based on the introduction of HFNOT at the hospital. Oxygen support in the pre-HFNOT group was provided by conventional oxygen therapy, non-invasive and invasive mechanical ventilation. HFNOT was used in patients who refused or were intolerant to non-invasive ventilation and during weaning from ventilation. The incorporation of HFNOT into the management of interstitial lung disease patients in this study resulted in lower in-hospital mortality, reduced requirement for sedation and analgesia and a lower incidence of discontinuation of oral intake. There was no difference in the incidence of complications (97).

Hypersensitivity pneumonitis is a form of interstitial lung disease resulting from the inhalation of small particulate antigens. Acute, subacute, and chronic forms have been reported which may resolve or progress to pulmonary fibrosis (98). Lycoperdonosis is a form of hypersensitivity pneumonitis associated with inhalation of spores from *Lycoperdon* spp. (puffball) mushrooms (99, 100). A recent abstract at the North American Congress of Clinical Toxicology reported the successful use of HFNOT and bronchodilators in the management of a child with lycoperdonosis. Corticosteroids were not required despite previous recommendations by some authors (101).

#### 3.1.4. Asthma

Acute exacerbation of asthma is a common cause of respiratory distress, with non-invasive oxygen support recommended in severe cases (102). However, the cold and dry air provided *via* conventional oxygen therapy modalities may potential exacerbate bronchoconstriction, promoted airway inflammation and impair mucociliary function (103). As such HFNOT has theoretical benefits in the management of severe asthma.

Retrospective studies of pediatric status asthmaticus showed significant improvements in vital parameters, serum pH and SpO_2_/FiO_2_ associated with HFNOT compared to conventional oxygen therapy (104, 105). A pilot study of a similar population supported these findings, demonstrating higher rates of pulmonary score improvement 2 h after initiation of HFNOT compared to conventional oxygen therapy (106). HFNOT has also been associated in decreased intubation rates for severe pediatric asthma (107). Despite these findings, improved patient outcomes have not been demonstrated (108). Further to this, some studies have raised concerns regarding delays in treatment escalation in patients receiving HFNOT (109). Clinical trials are needed to further investigate these findings.

#### 3.1.5. Carbon monoxide intoxication

Carbon monoxide competitively and reversibly binds to hemoglobin with 250 times greater affinity than oxygen resulting in a marked anemic hypoxia despite a normal P_a_O_2_ (110). Treatment involves provision of high FiO_2_ to compete with carbon monoxide for hemoglobin binding sites and reduce the half-life of carboxyhemoglobin (COHb) (1). HFNOT has recently been reported as an alternative treatment for carbon monoxide intoxication.

A prospective observational clinical study enrolled 33 adult patients presenting to two academic emergency departments with carbon monoxide intoxication (111). The primary objective of this study was to determine the mean half-life of COHb after HFNOT (FiO_2_ 100%, T 37°C, 60 L/min), which was found to be 36.8 mins. The investigators also assessed device tolerability and patient comfort. 11/33 patients requested a change in flow rate due to discomfort but then self-evaluated as very comfortable following rate adjustment (111). A subsequent retrospective study identified a similar COHb half-life following HFNOT (41.1 min) but this was found to not be significantly different to that in patients receiving conventional oxygen therapy (112). However, *post-hoc* analysis showed a significant difference in COHb levels between treatment groups at 60 and 90 mins.

#### 3.1.6. Procedural sedation

Oxygen desaturation, airway obstruction and apnoea are the most prevalent adverse events during procedural sedation (113). Risk factors for hypoxemia during procedural sedation include high ASA physical status, reduced cardiopulmonary reserve, obesity and prolonged procedural duration (114). Oxygen support *via* HFNOT has been evaluated during sedation for gastrointestinal endoscopy (115–117), bronchoscopy (118, 119), dental procedures (120, 121) and minimally invasive cardiovascular interventions (122, 123).

A recent meta-analysis evaluated the effected of HFNOT and conventional oxygen therapy during procedural sedation in adults and children, and included 4,121 patients in 19 randomized control trials. This found that HFNOT reduced the risk of hypoxemia and increased minimum oxygen saturation. Furthermore, the reduction in hypoxemia persisted regardless of the procedure, FiO_2_, risk-profile of the patient and mode of propofol administration (114).

#### 3.1.7. Post-extubation

Post-extubation respiratory insufficiency is a known complication following weaning from invasive mechanical ventilation and may progress to acute respiratory failure. Post-extubation acute respiratory failure results in extubation failure and reintubation (124). Conditions associated with post-extubation respiratory insufficiency include upper airway obstruction, decreased respiratory muscle function, atelectasis and increased work of breathing, and haemodynamic stability (125). Several studies have compared HFNOT and conventional oxygen therapy in relation to risk of reintubation. In low-risk patients, results of these studies have varied.

Zhu et al. attempted to quantify the benefits of HFNOT for patients after planned extubation (126). They evaluated results from 856 HFNOT patients and 852 patients who received conventional oxygen therapy in 10 studies. HFNOT was associated with reduced post-extubation respiratory failure, decreased respiratory rates and increased P_a_O_2_. However, no significant differences in reintubation rate, length of ICU and hospital stay, PaCO_2_, mortality or severe adverse events were identified (126). The unanticipated re-intubation rates may reflect heterogeneity in patient populations and treatment protocols. Additionally, some of the included studies contained conventional oxygen therapy groups for whom escalation in oxygen support included HFNOT and this may have confounded the results.

Another recent meta-analysis compared HFNOT and non-invasive ventilation in patients after extubation. Initial use of HNFOT in the post-extubation period was not inferior to non-invasive ventilation in regards to the probability of reintubation, treatment failure or mortality and was associated with a reduced probability of complications include cutaneous lesions and respiratory failure (125).

A multicentre randomized control trial compared HFNOT to HFNOT in combination with non-invasive ventilation in patients at high risk of extubation failure (127). Patients who successfully completed a spontaneous breathing trial after more than 24 h intubation were included. Those in the combination group commenced non-invasive ventilation immediately after extubation with a minimum duration of 12 h per day for the first 48 h. HFNOT was administered between non-invasive ventilation sessions in the combined group and continuously in the HFNOT sole treatment group. In the 641 patient who completed the study, reintubation rate at day 7 was significant higher in the HFNOT compared to when the therapies were combined. Of interest, the combination of HFNOT and non-invasive ventilation appeared to be more beneficial in patients with pre-extubation hypercapnia, defined as PaCO_2_ > 45 mmHg.

### 3.2. Complications and considerations

Complications of HFNOT are rare but include facial trauma, abdominal distension, aspiration, epistaxis and barotrauma. However, risk of these is lower than with other non-invasive ventilation modalities (77). A major concern during HFNOT is the risk of delayed intubation and mechanical ventilation in hypoxemic patients (74). A prospective study by Kang et al. (128) evaluated if delaying intubation until failure of HFNOT adversely affected patient outcome. 175 patients who failed HFNOT were categorized based on time at which intubation occurred; before (early) and after (late) 48 h of HFNOT. Patients intubated after 48 h had higher overall ICU mortality after propensity score adjustment and matching. However, the results of this study may have been affected by the lack of pre-determined criteria for intubation. Furthermore, the median duration of HFNOT prior to intubation in the late group was 126 h compared to 10 h in the early group (128).

HFNOT is generally considered to be well tolerated, particularly in comparison to other oxygen support modalities. In a small prospective, observational emergency room study, both patients (100%) and caregivers (82%) judged HFNOT to be more comfortable compared to conventional oxygen therapy (129). Patients also report less mouth dryness and improved breathlessness with HFNOT compared with facemask oxygen (63). However, patient discomfort is reported and may be associated with specific patient and treatment factors. *Post-hoc* analysis of the FLORALI trial reported higher patient discomfort after 1 h in patients failing HFNOT (130). Lower temperatures with full humidification was associated with lower discomfort regardless of flow rate in another study (131).

Prolonged placement of nasal cannulas for HFNOT may cause localized skin damage. Cutaneous and mucosal ulceration of the nose, nasal septum, frenulum and pinnae associated with friction between the skin and interface in prolonged HFNOT has been reported (132). Older patients and those with co-morbidities may be predisposed to such injuries (133). However, the risk of these lesion is significant less with HFNOT compared to non-invasive ventilation and CPAP (134, 135).

A retrospective study evaluated complications associated with HFNOT in a pediatric ICU over 1 year (136). Two children developed new pneumothoraxes, although neither could be specifically attributed to HFNOT-associated barotrauma and may have occurred secondary to the underlying conditions and other treatments of the patients. None of the six pre-existing pneumothoraxes worsened during HFNOT (136). Hodge and Prodhak (137) reported three life-threating occurrences of air leak syndrome associated with HFNOT in pediatrics—a 2-month old with bronchiolitis who developed a pneumothorax after HFNOT at 8 L/min, a 16-year old with non-respiratory illness who developed at pneumomediastinum after HFNOT at 20 L/min and a 22-month old with a sub-dural haematoma who developed a pneumothorax after HFNOT at 6 L/min. Pneumomediastinum, pneumothorax and subcutaneous emphysema has also been reported in an adult female treated with HFNOT (FiO_2_ 0.5, flow rate 40 L/min) for respiratory failure (138).

Tension pneumocephalus is a rare but potentially catastrophic complication of positive pressure ventilation and has been associated with HFNOT. Chang et al. reported a case of tension pneumocephalus is an adult with an unrecognized basilar skull fracture (139). HFNOT-associated tension pneumocephalus has also been reported in a pre-term neonate (140). Both events resulted in severe neurological sequelae from which the patients did not recover.

Contraindications for HFNOT include nasal, facial and airway abnormalities that may affect nasal cannula fit and device function or predispose to complications. Implicated conditions include epistaxis, basilar skull fractures, surgery to the nose and nasal obstruction (77).

### 3.3. Predictors of treatment failure

Despite the significant advantages of HFNOT in the management of acute respiratory failure, current literature indicates that around 30% of patients will fail treatment and require mechanical ventilation (74). In patients with acute hypoxemic respiratory failure, an increased heart rate after 1 h of HFNOT was associated with intubation (130). Hemodynamic instability has also been associated with HFNOT failure in cohort studies (141, 142). Other factors that have been associated with HFNOT failure include elevated SOFA score, thoracoabdominal asynchrony, significantly increased respiratory rate and poor oxygenation (143).

The ROX index was developed to predict success and failure of HFNOT (144). This index is the ratio of the pulse oximetry oxygen saturation over the fraction of inspired oxygen over the respiratory rate [(SpO_2_/FiO_2_)/RR]. The ROX index was evaluated in a prospective study of 157 pneumonia patients treated with HFNOT, with FiO_2_ set to maintain SpO_2_ > 92% and flow rate set at the clinicians' discretion. Treatment failure was assessed based on respiration, oxygenation and ventilatory parameters; criteria for intubation and mechanical ventilation were decreased Glasgow Coma Scale score, haemodynamic instability and persistent or worsening respiratory condition. A ROX index ≥ 4.88 measured after 12 h of HFNOT was significantly associated with a lower risk of mechanical ventilation (144).

A subsequent multicentre prospective observational cohort study by the same group validated the diagnostic accuracy of the ROX index (145). One hundred and ninety-one pneumonia patients treated with HFNOT were evaluated and the most specific cut-off to predict treatment failure and success were assessed. ROX ≥ 4.88 measured at 2, 6 or 12 h after HFNOT initiation was consistently associated with a lower risk for intubation. A ROX < 2.85, <3.47, and <3.85 at 2, 6, and 12 h of HFNOT initiation, respectively, were predictors of HFNOT failure. Patients who failed also demonstrated a lower increase in the values of the ROX index over the 12 h (145).

## 4. High-flow nasal oxygen therapy in veterinary patients

### 4.1. Canine

#### 4.1.1. Studies in healthy dogs

The Precision Flow^TM^ system was evaluated in a prospective study of 6 healthy client-owned dogs (23). One hundred percent oxygen was provided *via* conventional nasal oxygen cannula (100 ml/kg/min) or HFNOT (20 L/min and 30 L/min; equating to median flow rates of 0.7 L/kg/min and 1.1 L/kg/min with minimum and maximum rates of 0.55 L/kg/min and 1.7 L/kg/min) in a randomized order followed by a washout period. Blood gas values and end-expiratory transpulmonary pressures were assessed for each treatment after a 6-min acclimation period. This study demonstrated a significantly higher increase in baseline P_a_O_2_ between the delivery methods but no difference in P_a_O_2_ achieved using the HFNOT system at the different flow rates. No differences in transpulmonary pressure were identified between baseline and any of the treatment methods, or between any of the treatments, suggesting negligible generation of positive airway pressure. Although the minute ventilation was exceeded for all dogs at both flow rates, the small number dogs and marked variation in individual flow rates may have influenced the results.

Jagodich et al. evaluated the Optiflow^TM^/Airvo™ 2 system in a prospective randomized crossover study of eight healthy dogs (27). In this study 100% oxygen was provided *via* conventional nasal cannula or HFNOT at a range of flow rates for each system. Additionally, the effects of HFNOT were compared between sedated and unsedated dogs. Vital parameters, airway pressure and gas values, respiratory scores and tolerance were recorded for each treatment and flow rate. Airway pressures generated by each system were comparable at equivalent flow rates. However, only HFNOT at flow rates of 1–2 L/kg/min was able to maintain positive airway pressure and achieve CPAP in the majority of dogs. Furthermore, both inspiratory and expiratory airway pressures significantly increased with increases in flow rate. While undulations in airway pressure wave form were identified in panting dogs, expiratory pressure remained positive at high flow rates (27).

Harduin et al. prospectively evaluated the impact of gas flow rate and temperature on tolerance of 12 dogs receiving HFNOT (Optiflow^TM^/Airvo™ 2 system) during recovery from anesthesia (25). Four treatment conditions (2- or 4-times estimated V_T_; 0.8 L/kg/min or 1.6 L/kg/min) and 31°C or 37°C) per dog were randomly applied after placement of a nasal catheter immediately after extubation. Vital parameters, level of sedation and tolerance were assessed at initiation and after 10 mins for each treatment. This study showed no effect of flow rate or temperature on vital parameters or tolerance, and overall good tolerance in this group of dogs. A single dog did not tolerate the first treatment (4 × V_T_ at 37°C) but tolerated subsequent treatments. It could not be determined if this were associated with the specific protocol or dysphoria associated with emergence from anesthesia (25). These findings in dogs conflict with findings in humans in which lower set temperatures (31°C vs. 37°C) have been associated with increase patient comfort and tolerance (131).

#### 4.1.2. Clinical applications

##### 4.1.2.1. Hypoxaemic respiratory failure

All canine studies of acute hypoxemic respiratory failure at this time include a heterogeneous population and as such suitability and response to treatment of specific conditions cannot be determined. Notwithstanding, the majority of cases in all studies are associated with pneumonia.

Keir et al. first described the use in HFNOT in veterinary patients in six dogs with primary pulmonary hypoxemia (26). In this retrospective study four dogs had aspiration pneumonia, one dog had eosinophilic pneumonopathy with concurrent aspiration pneumonia and one had pulmonary contusions. All dogs were transitioned to HFNOT following failure of conventional oxygen therapy to maintain adequate oxygenation. A significant increase in mean PaO_2_ (61.85 mmHg on COT to 133.75 mmHg on HFNOT) was achieved in association with a significant increase in oxygen flow rate (122 ml/kg/min on COT to 688 ml/kg/min on HFNOT). Overall, four of the dogs had resolution of their hypoxemia following HFNOT (26).

A prospective pilot study of the effectiveness and tolerance of HFNOT therapy in 11 dyspnoeic dogs included nine dogs with primary pulmonary hypoxemia (30). Dogs were included if they were transitioned to HFNOT after failing to stabilize after 30 mins of treatment with medical therapy and conventional oxygen therapy; and assessed for 60 mins after the initiation of HFNOT. Underlying conditions in patients included five cases of aspiration pneumonia, and one each of cardiogenic pulmonary oedema, non-cardiogenic pulmonary oedema, leptospirosis and pulmonary hemorrhage secondary to trauma. After 60 minutes of treatment with 100% FiO_2_ at a flow rate calculate to match minute ventilation there was a significant increase in mean PaO_2_ and resolution of hypoxemia in 5/8 dogs with PaO_2_ < 80 mmHg prior to initiation of HFNOT. Although 6/11 dogs had a decrease in respiratory rate only 2/11 were classified as not in respiratory distress base on the authors' criteria (RR < 40 breaths/min). Overall, HFNOT was well tolerated in this group of dogs (30). 6/11 dogs in this study died as result of cardiac arrest or euthanasia due to deteriorating clinical condition. Of the non-surviving group, 5/6 met criteria for intubation within 24 h of admission (30).

A second prospective study has also evaluated HFNOT in acute hypoxemic respiratory failure in 22 dogs (28). Dogs were included in the study if they had no improvement in oxygenation (*n* = 11), work of breathing (*n* = 10) or both (*n* = 1) after 30 mins of treatment with conventional oxygen therapy. Underlying conditions included pneumonia (*n* = 7), inflammatory disease (*n* = 3), pulmonary arterial hypertension (*n* = 3) and congestive heart failure (*n* = 2). Patients were not included if immediate intubation/mechanical ventilation was indicated, unless this treatment was declined by the owners. Vital parameters, blood gases, patient tolerance, work of breathing and level of sedation were evaluated at baseline, 30 mins, 60 mins and then every 6 h after initiation of HFNOT. Patients remaining on HFNOT beyond 1 h did so for a median for 18 h, however, analysis was only performed on data collected for the first 7 h of treatment so as to limit the influence on patient condition of other concurrent treatments. The majority of dogs in this study received 100% FiO_2_ at the initiation of treatment, although 3 were started at lower levels; the median FiO_2_ reduced significantly over all of the following time points. The median flow rate was 1 L/kg/min for the first 7 h for which data was evaluated. Significant improvements in dyspnoea score and oxygen saturation were identified at all time points compared to baseline with a moderate correlation between HFNOT flow rate and PaO_2._ There was no significant difference if PaCO_2_ between conventional oxygen therapy and HFNOT treatment, although there was a moderate correlation between PaCO_2_ and HFNOT flow rate. 6/22 dogs in this study were intubated and ventilated after starting HFNOT and an additional 6/22 were euthanized due to required escalation of therapy. Overall, 10/22 dogs survived to discharge, including 8/22 who avoided intubation and mechanical ventilation (28).

##### 4.1.2.2. Post-extubation

The use of post-extubation HFNOT in brachycephalic dogs has also be investigated (29). Jagodich et al.'s prospective study included five brachycephalic dogs with signs of upper airway obstruction in the immediate post-anesthetic period. Four dogs were receiving supplemental oxygen and 4/5 were commenced on HFNOT for non-hypoxemic signs of brachycephalic obstructive syndrome. Patients were treated with sedation at the clinicians' discretion and commenced on HFNOT at 0.5–1.5 L/kg/min and variable FiO_2_ depending on their oxygenation. Dyspnoea scores and respiratory rates improved over time with stable normoxemia despite decreasing FiO_2_ and flow rates. HFNOT was able to be discontinued in <12 h in 3 dogs and all dogs survived to discharge without requirement of reintubation (29). Unfortunately, the design of this study did not allow for comparison with standard treatment.

##### 4.1.2.3. Carbon monoxide poisoning

A case report by Gazsi et al. compared the use of mechanical ventilation and HFNOT in two dogs with carbon monoxide poisoning following progression of clinical signs despite conventional nasal oxygen therapy (146). One dog received HFNOT at 100% FiO_2_ at 1 L/kg/min for 4 h. After this time co-oximetry indicated marked improvement in FCOHb and oxygen supplementation was discontinued. Interestingly, the dog treated with mechanical ventilation had similar improvements in FCOHb and was weaned off the ventilator in the same time period. The calculated half-life of FCOHb was 167 min and 150 min in the dogs treated with HFNOT and IPPV, respectively.

#### 4.1.3. Complications

Few complications have been identified in canine patients at this time, the majority of which have been subclinical and self-limiting.

An experimental crossover study of HFNOT in health dogs demonstrated a significant increase in PaCO_2_ in dogs following heavy hydromorphone/dexmedetomidine sedation and HFNOT, a finding not identified in unsedated dogs or dogs treated with conventional oxygen therapy in the same study (27). These findings were not supported in another study of six sedated healthy dogs in which no significant differences in PaCO_2_ were identified between baseline for any oxygen delivery method, including HFNOT delivered up to 1.75 L/kg/minute (23). These differing findings between the two groups of healthy dogs may have been, in part, influenced by the sedation protocols used and level of sedation achieved (23, 27).

A mild but significant increase in PaCO_2_ was identified in dogs with primary hypoxemia following initiation of HFNOT (26). Moderate-to-severe hypercapnia was also observed in 3/5 brachycephalic dogs treated with HFNOT in the post-anesthetic period. The relative role of sedation, HFNOT and underlying condition in the development of hypercapnia in these patients could not be further elucidated (29). These findings were not replicated in the HOT-Dog study (30).

Aerophagia was noted on thoracic radiographs in 8/8 healthy dogs receiving HFNOT in one study (27). Gastric distension was also identified on radiographs in 1/6 healthy dogs receiving HFNOT another study (23). In the study of post-anesthesia HFNOT in brachycephalic dogs, one dog experienced severe aerophagia requiring orogastric intubation. This dog experienced no further complications despite remaining on HFNOT for an additional 19 h (29).

No cases of air leak syndrome have been identified in canine HFNOT patients. However, in the study by Keir et al. one dog experienced persistence of a pre-existing pneumothorax which only resolved following discontinuation of HFNOT (26).

### 4.2. Equine

Floyd et al. have recently described the use of HFNOT (Optiflow^TM^/Airvo™ 2) with a modified interface in foals < 36 h old (24). This retrospective study reports the use of HFNOT in foals with a variety of clinical diagnoses. Treatment was initiated with a target flow rate of 40 L/min and variable FiO_2_ based on arterial blood gas analysis. No significant improvement in oxygenation with treatment was identified although not all foals were hypoxemic prior to initiation of HFNOT. However, improvements if respiration pattern and decreased respiratory rates were observed (24).

Overall, 10/14 foals survived to discharge and HFNOT was considered well tolerated in all patients. No significant complications were observed in this group although two foals required escalation of therapy (mechanical ventilation) and HFNOT had to be discontinued in two foals due to excessive activity. Unlike in many canine studies, no significant increase in mean PaCO_2_ was observed, and conversely, a mild but insignificant decrease in PaCO_2_ was observed in many foals (24).

### 4.3. Feline

A single Letter to the Editor briefly reports the successful clinical use of HFNOT in cats (147). The authors report the need for sedation to enable the appropriate placement and securing of the nasal cannula but that treatment has been otherwise well tolerated and may be considered as an alternate treatment to CPAP generated by helmet (12).

## 5. Clinical application of high flow nasal oxygen therapy

No specific veterinary protocols are available for HFNOT. However, increasing clinical experience and published investigations in animals, in combination human guidelines, has enabled repeatable and successful use of HFNOT (Table 2).

**Table 2:** A quick guide to HFNOT set up in veterinary patients.

**Sedation**
• Only if required • Base on patient assessment and comorbidities • Butorphanol and/or α_2_-agonists commonly use
**Nasal prongs**
• Occlude < 50% opening of nares • Suture to keep in place
**Initial settings**
• FiO_2_ 100%
• Flow rate 1–2 L/kg/min
• Temperature 37°C
**Monitoring**
• Continuous ECG • RR, respiratory effort SpO_2_ every 1–2 h • PaO_2_ and PaCO_2_ at least every 12 h
**Weaning**
• Reduce FiO_2_ by 5–10% every 1–2 h based on SpO_2_ • Reduce flow rate after stable RR and SpO_2_ on 40% FiO_2_ for 12–24 h • Consider discontinuing when FiO_2_ < 30–40%
**Indications for escalation**
• No improvement after 1–2 h of high FiO_2_ and rate • Persistent increased work of breathing • Hypercapnia/hypoventilation

### 5.1. Nasal prongs

Nasal prongs specific for HFNOT are designed to optimize device function and patient comfort. Selection should be based on the recommendations of the device manufacturer. The prongs should occlude ~50% of the opening to the nares to facilitate generation of desired airway pressures while minimizing resistance to exhalation (23, 28). In very small patients, placement of a prong into a single nares has been advocate to create overall 50% nasal occlusion (28). Instillation of local anesthetic into the nares prior to placement of nasal prongs may facilitate placement and promote tolerance (28).

**Figure 3:**
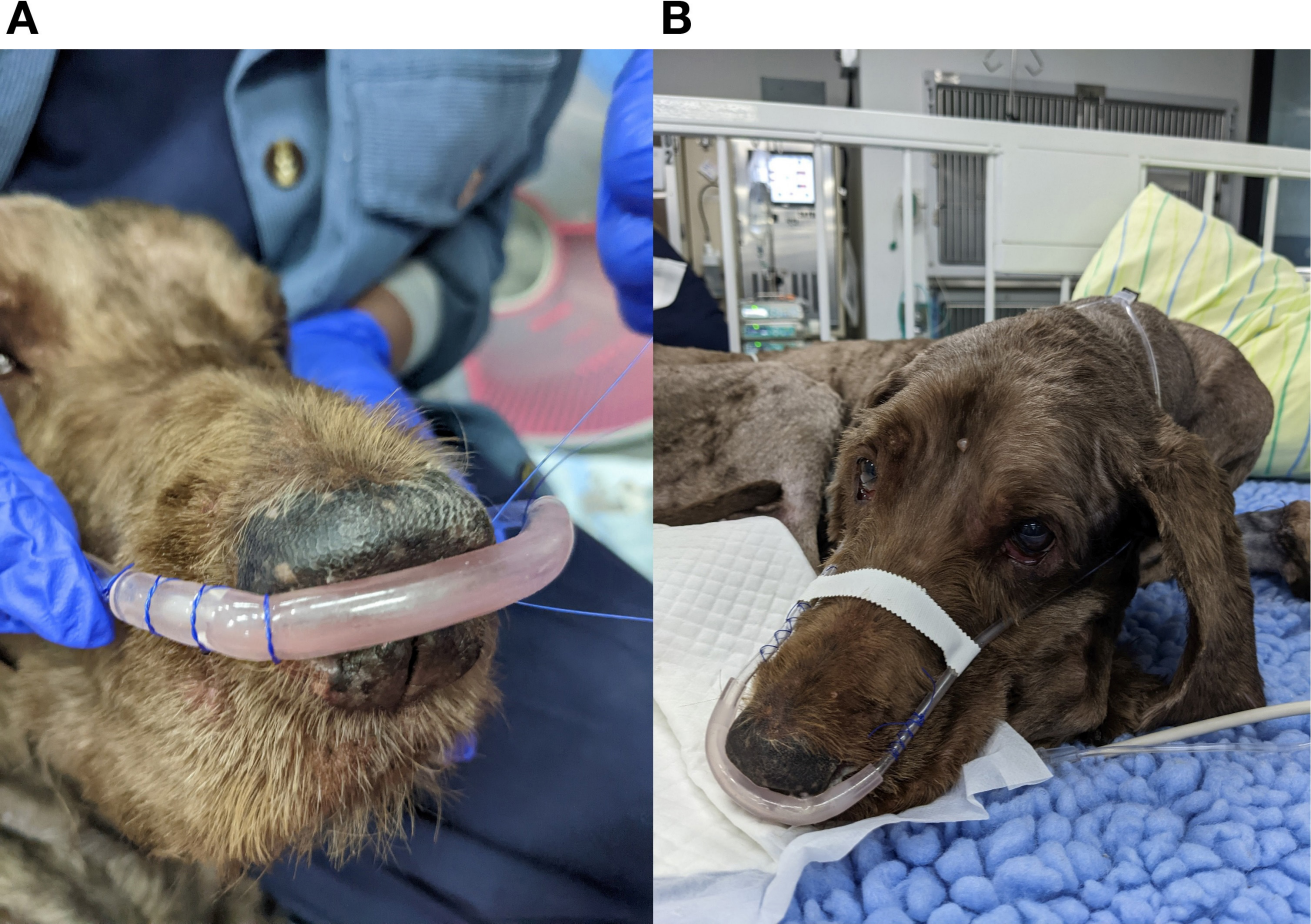
Nasal prongs often need to be secured with sutures at the margins of the nares **(A)**. Taping over the muzzle keeps the interface in place in dolichocephalic breeds **(B)**.

As nasal prongs are designed for humans some modification or additional fastening is required in veterinary patients. A variety of suturing and taping techniques have been used, although suturing of the prongs to the margins of the nares appears to be most effective in keeping the prongs in position and limiting dislodgement (Figure 3). The use of modeling clay to improve the nasal prong interface in dogs has been advocated by some authors (27).

Floyd et al. (24) described a nasal prong modification to facilitate HFNOT in foals. Thoracic drains (24 Fr) measured to level of the medial canthus are connected to a Y-adaptor and then directly to the breathing circuit. The nasal catheters are then sutured to the nostrils. Smaller diameter nasal catheters were unable to deliver higher flow rates (24).

### 5.2. Device set-up

Patients should initially receive 100% FiO_2_ while stabilization occurs. This may then be titrated down based on patient oxygenation, aiming to maintain an SpO_2_ > 95% or PaO_2_ > 80 mmHg (30). Timely reduction of FiO_2_ to <60% is recommended due to concerns for oxygen toxicity associated with prolonged administration of high concentrations of oxygen (2, 30).

Flow rates should be commenced at 1–2 L/kg/min or calculated to match the patients minute ventilation (respiratory rate × V_T_). A CPAP effect in dogs occurs at flow rates of 1–2 L/kg/min and these rates are generally well tolerated (27). A study assessing the implementation of a HFNOT protocol in the human pediatric intensive care patients demonstrated more rapid weaning, decreased failure rates and possibly decreased rate of escalation to positive pressure ventilation in patients initially treated with higher flow rates. These patients also received lower initial FiO_2_ and had a shorter length of stay in hospital despite longer duration of HFNOT (148). However, higher flow rates may result in patient discomfort and aerophagia (27).

The temperature is initially set at 37°C but may be adjusted based on patient body temperature and comfort. In humans, lower set temperatures (31°C vs. 37°C) have been associated with increased patient comfort and tolerance (131). No difference in tolerance was identified in healthy dogs administered HFNOT at different flow rates and temperatures (25).

### 5.3. Weaning

No weaning protocols are available for veterinary patients and human guidelines vary at this time. However, it is reasonable to consider similar criteria as are used for weaning from other methods of oxygen support—improvement in underlying condition, decreased device settings, high likelihood of coping with de-escalated therapy (e.g., conventional oxygen therapy). Patients with stable respiration and oxygenation at <500 ml/kg/min FiO_2_ < 40% will likely meet these criteria.

FiO_2_ should be reduced prior to reducing the flow rate. Reductions of 5–10% FiO_2_, followed by reassessment in 1–2 h is recommended (6). In human adults, maintenance of stable respiratory parameters at 40% FiO_2_ should be achieved prior to flow weaning. In neonates, it has been recommended that FiO_2_ be reduced to 30% prior to reductions in flow rate (149).

Respiratory rate, FiO_2_ and work of breathing should be stable for 12–24 h before commencing flow reduction (149). Reductions should be gradual and proportionate to patient size. Patients should be weaned onto low flow nasal oxygen which may be provide *via* HFNOT prongs and bubble humidifier.

### 5.4. Escalation

Based on human recommendations and clinical studies, escalation of treatment is recommended when there is no clinical improvement in 1–2 h despite high flow rates and FiO_2_ (6). Failure to maintain adequate oxygenation despite HFNOT or persistent increased work of breathing are indication for positive pressure ventilation. Due to limitation in availability and utility of non-invasive ventilation in veterinary patients, mechanical ventilation is often the next step in escalation of therapy. Mechanical ventilation is also indicated in cases of severe or progressive hypoventilation (16).

### 5.5. Sedation

Many studies have reported the use of sedation to facilitate initiation and/or maintenance of HFNOT in veterinary patients, although this is not required in all patients (25, 26, 28, 30, 147). Sedation protocols should be based on individual patient factors and assessment, however, the use of butorphanol and/or α_2_-agonists is often reported (150, 151).

## 6. Discussion

HFNOT provides a modality of high flow oxygen delivery allowing predictable FiO_2_ delivery with additional benefits previously only available with non-invasive and invasive ventilation. Its use has traditionally been focused on the management of acute hypoxic respiratory failure; however, an increasing range of potential indications is being reported and investigated in human and veterinary medicine.

Pneumonia is the most commonly reported indication for HFNOT in dogs and adult humans (26, 28, 30, 77). Until recently euthanasia and mechanical ventilation have been the only available options for veterinary patients with pneumonia that deteriorate despite conventional oxygen therapy (152, 153). HFNOT provides an alternative to these. Its use in veterinary patients appears promising, however, further investigations with appropriate patient selection are indicated.

Oxygen supplementation is recommended for the management of stage C myxomatous mitral valve disease in dogs and cardiomyopathy in cats based on expert opinion and is typically provided *via* oxygen cages or nasal cannulas (154, 155). Short-duration positive pressure ventilation has been shown to result in good outcomes for dogs and cats with acute congestive heart failure refractory to conventional management (156). The authors of this study postulated that mechanical ventilation in these patients was effective due to the same mechanisms that have been identified in humans—improved oxygenation, alveoli recruitment, improved lung compliance and reduced afterload, although this was not specifically assessed (156).

HFNOT has been advocated in the management of pulmonary oedema secondary to acute heart failure in humans (157). Improvement in oxygenation in these patients is attributed to provision of high FiO_2_, recruitment of collapsed alveoli and decreased preload (74, 157). A small number of dogs with congestive heart failure have been include in heterogeneous populations in previous studies, however the utility and efficacy in these patients has not been established (26, 28, 30). Whilst likely to be similarly effective and by the same mechanisms of action as in humans, the use of HFNOT in veterinary patients requires further investigation.

Asthma is a common inflammatory condition of the lower airways in cats believed to have an allergic etiology (158). Acute asthma attacks are managed with oxygen supplementation and administration of bronchodilators (159). Based on the human literature, HFNOT presents theoretical benefits in the provision of oxygen support in acute exacerbations of feline asthma. However, reports of the use of HFNOT in cats are too scarce to evaluate its feasibility (147).

The interstitial lung diseases of veterinary patients are poorly understood but generally carry a similarly poor prognosis to those of humans (160, 161). Pulmonary fibrosis in West Highland white terriers is the most thoroughly investigated in this group of disease, however there remains no effective treatment. Affected dogs may experience acute worsening of their respiratory function and signs due to bacterial pneumonia or acute exacerbation of the idiopathic pulmonary fibrosis (162). Despite empirical treatments, the short-term mortality rate in dogs is similarly poor to that of humans (93, 162). Long-term oxygen therapy is recommended in humans with pulmonary fibrosis but is not feasible in dogs (163). However, as with humans, HFNOT may be considered for intermittent and short-term treatment in dogs with acute exacerbations of pulmonary fibrosis.

Lycoperdonosis is a rarely reported reversible interstitial lung disease in dogs with a variable prognosis (164–166). No treatment protocols exist for this condition; however, the provision of oxygen support is indicated. HFNOT therapy has been reported to be effective in humans and may be considered in future veterinary cases, particularly in patients that do not respond to conventional oxygen therapy (101).

There are currently no protocols for HFNOT in veterinary species and recommendations are based on those for humans, a small number of veterinary cohort studies and practical experience (24, 26–28, 30). Further investigations are warranted to optimize protocols for individual species and disease conditions. Greater understanding of the role of the individual mechanism of HFNOT in improving respiration and oxygenation in different conditions will help to define the ideal settings for these. The effect of panting (open-mouth breathing) on the FiO_2_ delivery and PEEP generation in veterinary patients also warrants further investigation.

HFNOT is a non-invasive form of high flow oxygen support. Although it is considered relatively low risk with a low incidence of complications, rare serious and potentially catastrophic events are reported in humans (137, 139, 140). Consideration and identification of risk factors for adverse consequences should be made in veterinary patients to minimize the incidence of complications.

Hypercapnia is the most commonly reported complication in veterinary HFNOT studies (26, 27, 29). This has been attributed to the effects of sedation and increased resistance to exhalation associated with continuous high inspiratory flow rates by some authors (27). A role for alveolar overdistension may also be consider. During HFNOT V_T_ has been shown to increase proportionally with gas flow, independent of respiratory rate (167–169). A similar proportionally related increase in PaCO_2_ relative to flow rate has also be identified in hypoxemic dogs treated with HFNOT (28). Iatrogenically increased V_T_ has been associated with alveolar overdistension using other modalities (170) and HFNOT may induce alveolar overdistension in some individuals with an increase in risk identified in patients with low potential for alveolar recruitment (171). Over-distension leads to increased areas with high ventilation-perfusion inequality and dead space, causing CO_2_ retention (172, 173). Whilst levels of hypercapnia were not clinically significant in the animals in these studies, further investigation into the use of HFNOT in hypoventilation-induced hypoxemia is indicated.

Trauma is a common cause for admission in veterinary hospitals, with severe trauma occurring in approximately one third of these patients (174). Pulmonary contusions occur commonly in dogs and cats that have sustained blunt force trauma and the majority require oxygen support in the recovery period (175, 176). In 143 dogs with pulmonary contusion secondary to motor vehicle accidents 47% had pneumothoraxes, 10% had pulmonary bullae, 6% had pneumomediastinum and 13% had rib fractures (175). Iatrogenic pneumothorax is known to occur in dogs ventilated for pulmonary contusions secondary to trauma with concurrent thoracic pathology associated with increased risk (177). HFNOT generates positive airway pressure and has been associated with the development of pneumothoraxes in children (137). Although the development of pulmonary air leaks have not been reported in veterinary patients associated with HFNOT, a pre-existing pneumothorax in a dog with pulmonary contusions failed to resolved until HFNOT was discontinued (26). Care should be taken in evaluation of concurrent thoracic pathology and for iatrogenic causes of respiratory deterioration in patients with pulmonary contusions being treated with HFNOT.

Trauma in dogs and cats typically results in injury to multiple body regions (178, 179). In one study, 26% of dogs and 42% of cats had head injuries identified on physical examination after a traumatic event (179). Tension pneumocephalus has been reported secondary to HFNOT in a man with an undiagnosed skull fracture (139). Due to the high incidence of polytrauma in veterinary patients, consideration should be made of the potential for skull fractures in patients prior to commencing HFNOT for pulmonary contusions.

In the small number of available clinical veterinary studies evaluating HFNOT in the treatment of acute hypoxic respiratory failure, 44% (17/39) of dogs did not require escalation of therapy and survived to discharge (26, 28, 30). Due to the heterogeneity of the study populations and limited cases numbers insufficient data is currently available to determine accurate predictors of treatment success and failure. Development of a scoring system similar to the ROX index through large retrospective or prospective studies may aid in patient selection and the early determination of patient prognosis (144, 145).

## Author contributions

JW and IK contributed to conception of the article and manuscript review and wrote sections of the manuscript. JW performed the literature review and wrote the first draft of the manuscript. All authors read and approved the submitted version.
